# Investigating the feasibility and effectiveness of bacterial culture in negative pressure wound drainage fluid

**DOI:** 10.3389/fmed.2024.1479698

**Published:** 2024-11-21

**Authors:** Junjie Wu, Zhengqi Chang

**Affiliations:** Department of Orthopedics, 960th Hospital of PLA, Jinan, China

**Keywords:** spinal infection, negative pressure drainage, bacterial culture, positive rate, drug sensitivity result

## Abstract

**Purpose:**

To analyze the feasibility and effectiveness of bacterial culture in negative pressure wound drainage (NPWD) fluid in patients with Pyogenic Vertebral Osteomyelitis (PVO).

**Methods:**

A retrospective analysis was performed on 17 patients with PVO who were treated with negative pressure drainage at the Department of Orthopedics in our hospital from January 1, 2018 to December 31, 2021. Data was obtained while the patients were in the hospital, including 12 males and 5 females with an average age of 57.71 ± 9.93 years. After applying the negative pressure drainage technique, the drainage fluid was collected and a bacterial culture was performed. The positive rate of bacterial culture in the drainage fluid was recorded. Comparing the positive rate of specimen culture obtained by this surgical method with other methods reported in the literature, the effectiveness of negative pressure drainage in the treatment of PVO was analyzed.

**Results:**

All patients were placed with negative pressure sponge during operation and underwent continuous negative pressure aspiration after operation. The average of total drainage volume was 186.47 ± 29.44 mL. The drainage fluid was successfully retained for bacterial culture, and the results of bacterial culture were negative in 3 patients. Pathogenic bacteria were successfully obtained from negative pressure drainage fluid in 14 cases, with a positive rate of 82.4% (14/17).

**Conclusion:**

Using negative pressure drainage to retain drainage fluid for bacterial culture can significantly increase the positive rate, which is helpful for the diagnosis of PVO and rational antibiotic treatment.

## Introduction

Spinal infections are a common type of bone infection, accounting for approximately 3% of all bone infections (1). The prevalence of spinal infections is on the rise due to factors such as increased life expectancy, chronic illnesses, antibiotic usage, and spinal surgeries (2). In China, the aging population, improved diagnostic techniques, and heightened susceptibility have contributed to the increasing occurrence of spinal infections (3). Symptoms of spinal infections typically include low back pain, swelling, and other discomforts. If left untreated, these infections can result in nerve damage or even death (4). However, spinal infections are often misdiagnosed due to their subtle onset and atypical symptoms, leading to an average diagnosis time of 2–6 months (5). In cases where a spinal infection is confirmed, some patients may require surgical intervention, especially if they have nerve damage, spinal instability, or spinal deformities (6). Proper administration of antibiotics is essential for both conservative and surgical treatments, with the selection of antibiotics based on bacterial culture and drug sensitivity. For non-specific vertebral infections (Pyogenic Vertebral Osteomyelitis), the identification of pathogenic microorganisms through bacterial culture and the effective use of sensitive antibiotics are crucial priorities in clinical management.

Due to the low rate of positive blood culture results in patients with abscesses, puncturing to obtain pus for culture and identification of pathogenic bacteria is the most reliable method of diagnosis and treatment (7). However, primary spinal infections are located deep, with few skin sinus openings, and large abscesses tend to spread along the bilateral psoas major muscles, making it more difficult to obtain specimens than with other superficial infections. Common techniques for obtaining specimens include B-ultrasound or CT guided puncture and drainage surgery, intervertebral foramen endoscopic lesion clearance surgery, and incision lesion clearance surgery (8). Nevertheless, in some cases, the positive rate of pus culture is still not satisfactory due to the prolonged use of antibiotics.

We implemented Negative Pressure Wound Drainage (NPWD) technology to treat deep infection after spinal surgery, with successful results and a higher rate of bacterial culture (9). We then extended the use of NPWD to treat primary spinal infection, using small incisions to place negative pressure drainage for continuous drainage of secretions from patients with Pyogenic Vertebral Osteomyelitis (PVO). This method proved to be effective and safe, with a higher rate of bacterial culture and satisfactory clinical results when compared to other surgical methods reported in the literature.

## Materials and methods

A retrospective study was carried out on 57 patients diagnosed with pyogenic vertebral osteomyelitis (PVO) at our clinic from January 2018 to December 2021. The study focused on individuals who underwent debridement surgery and subsequently received continuous negative pressure wound therapy. Seventeen PVO patients were included in the study, consisting of 12 males and 5 females, with an average age of 57.71 ± 9.93 years. The cases consisted of one thoracic infection, one combined thoracic and lumbar infection, six lumbar infections, four combined lumbar and psoas major muscle infections, two infections after posterior cervical internal fixation, and three infections after posterior lumbar internal fixation. Preoperative antibiotic use was recorded in 76.5% of cases. Negative pressure drainage technology was utilized, and drainage fluid was collected for bacterial culture to determine the positivity rate. Subsequent internal fixation surgery was considered based on infection control outcomes.

**Figure 1:**
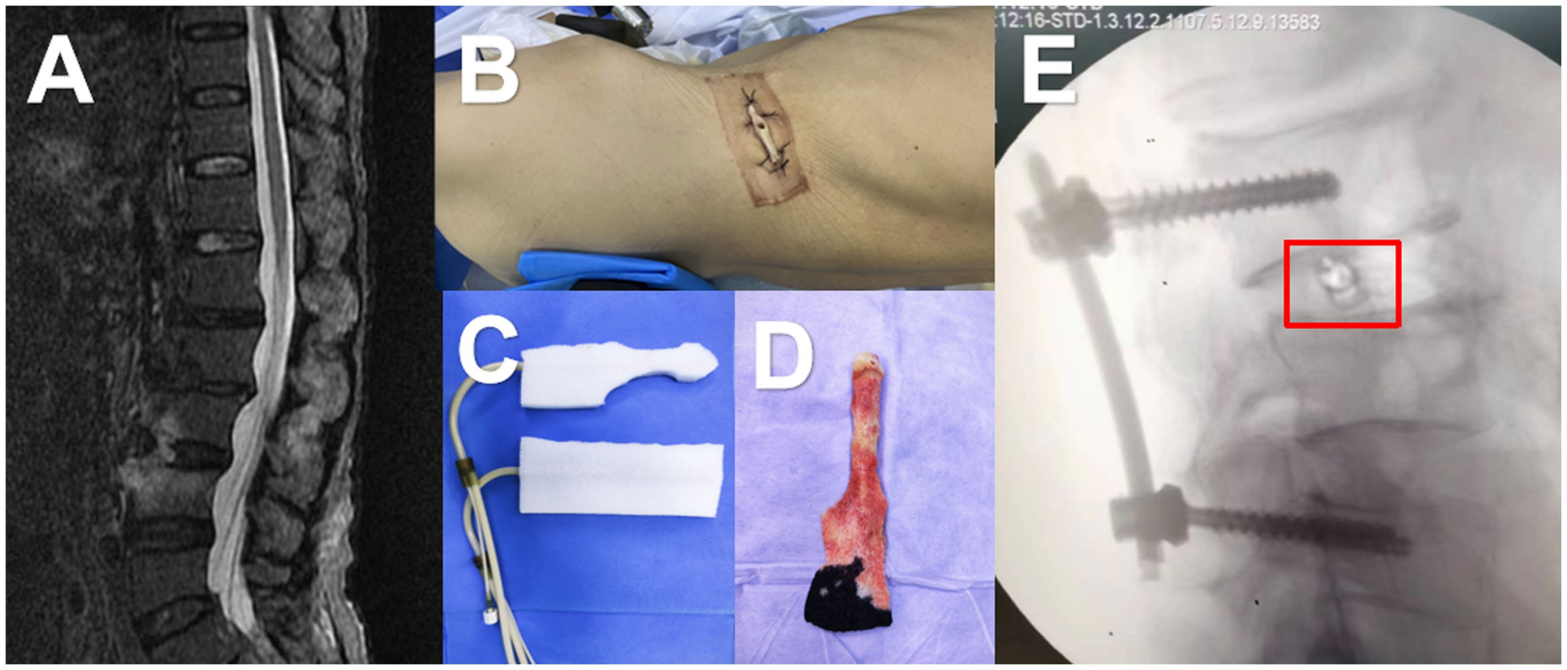
A 68-year-old male was admitted to the hospital with a month-long history of low back pain and fever. **(A)** MRI revealed increased signals on the anterior and inferior margins of the L3 and L4 vertebrae, as well as T2 liposuppressive images in the L3/4 space. **(B)** The patient was then placed in a lateral position and underwent a small incision debridement. **(C,D)** A negative pressure drainage sponge was inserted into the intervertebral space, and the negative pressure was connected. **(E)** Intraoperative fluoroscopy showed that the sponge was in the correct position (marked in red box).

**Figure 2:**
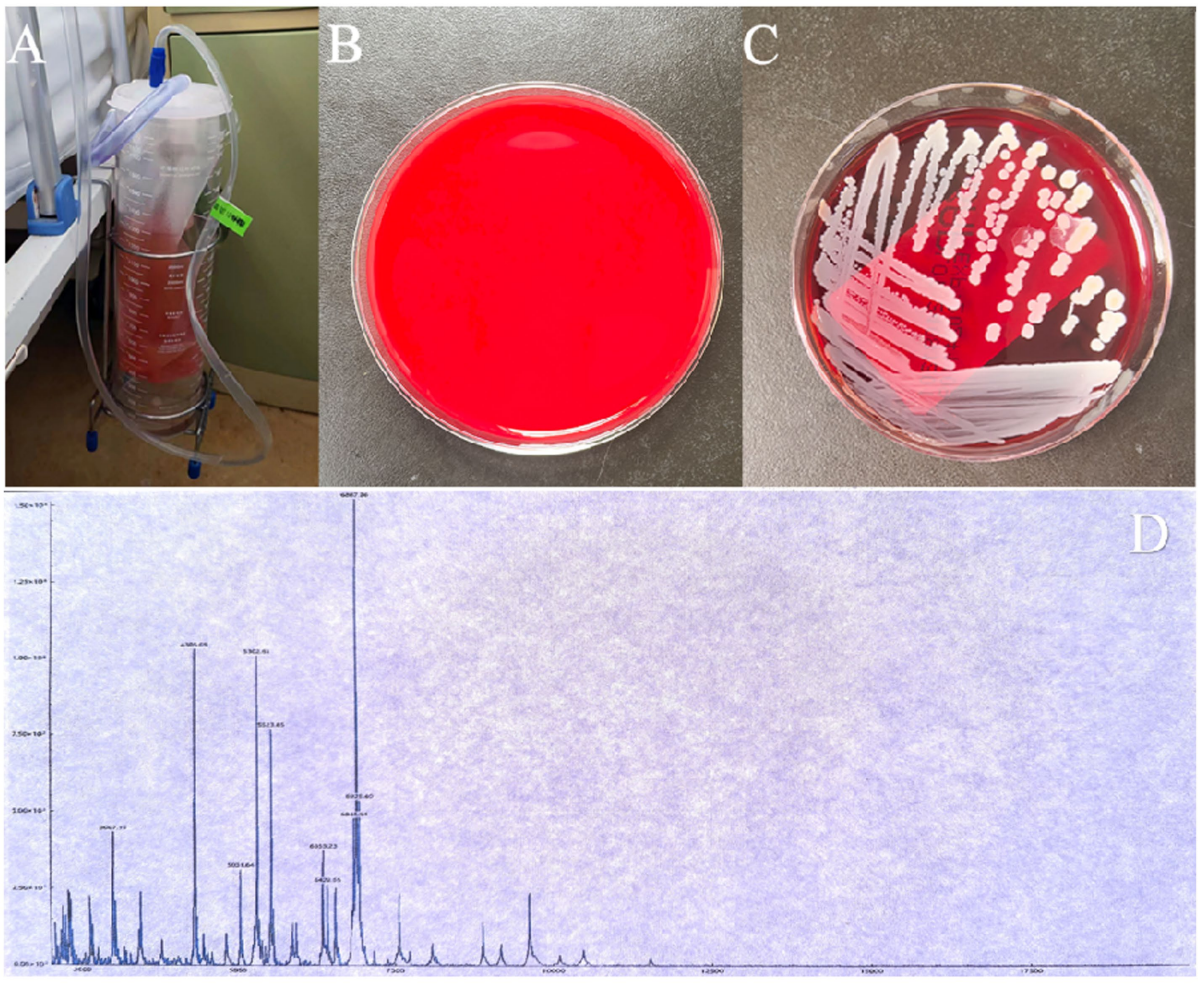
**(A,B)** The negative pressure drainage bottle and tube are replaced after 24 h, and the fluid is collected for bacterial culture. **(C)** *Staphylococcus aureus* is identified in the bacterial culture of the drainage fluid. **(D)** *Staphylococcus aureus* is confirmed by mass spectrometry, with an identification score of 2.29 (scores below 1.7 are not reliable, scores between 1.7 and 2.0 indicate possible genus-level identification, and scores equal to or greater than 2.0 indicate possible species-level identification).

After administering general anesthesia, the surgical site is prepared by disinfecting and covering it with tissue. The procedure begins with the use of a percutaneous screw fixator, followed by a small incision to remove the affected area through the intervertebral foramen. Tissue samples are taken for bacterial culture and pathological examination. Physiological saline is used to flush out the infected area during the surgery, and an NPWD sponge is placed to complete the procedure (Figure 1). A negative pressure drainage bottle is replaced 24 h later to maintain sterility and avoid contamination, and the fluid is collected for bacterial culture. Bacterial culture is performed according to the National Clinical Laboratory Operating Regulations. Specimens are inoculated onto various agar plates and incubated in a carbon dioxide incubator at 35°C for 16–20 h. The bacteria are then isolated, purified, and identified using a fully automated microbiological analyzer (Figure 2).

## Results

All patients had negative pressure sponges placed during surgery, followed by postoperative continuous negative pressure suction. The negative pressure drainage fluid was collected 24 h after surgery, and 5-10 mL was taken for bacterial culture. The total drainage volume was an average of 186.47 ± 29.44 mL. Out of the 17 patients, 14 (82.4%) had pathogenic bacteria in their drainage fluid, including 4 *Staphylococcus aureus*, 3 *Staphylococcus epidermidis*, 2 *Streptococcus viridans,* 1 *Streptococcus pharyngitis*, 1 *Escherichia coli*, 1 *Enterobacter cloacae*, 1 *Klebsiella pneumoniae*, and 1 *Candida tropicalis*. The remaining 3 patients had negative bacterial culture results (Table 1).

**Table 1:** shows general information of patients and bacterial culture results.

Number	Sex	Age	Site of lesion	Drainage volume (ml)	Prior operations	Pathogenic bacterium from NPWD	Pathogenic bacterium from tissue
1	F	50	L	190	N	/	
2	M	60	L, PM	225	N	*Escherichia coli*	*Escherichia coli*
3	F	62	L	225	N	*/*	
4	M	46	L	245	N	*Candida tropicalis*	*Candida tropicalis*
5	M	76	L	170	N	*/*	
6	F	46	L	190	N	*Klebsiella pneumoniae*	*Klebsiella pneumoniae*
7	F	61	T	215	N	*Streptococcus viridans*	*Streptococcus viridans*
8	F	57	L	185	N	*Streptococcus viridans*	*Streptococcus viridans*
9	M	53	L, PM	150	N	*Streptococcus pharyngitis*	
10	M	77	L, PM	190	N	*Staphylococcus aureus*	
11	M	59	T, L	155	N	*Enterobacter cloacae*	
12	M	52	C, S	170	N	*Staphylococcus epidermidis*	
13	M	68	L, S	215	N	*Staphylococcus epidermidis*	
14	M	55	L, S	135	N	*Staphylococcus epidermidis*	*Staphylococcus epidermidis*
15	M	42	L, PM	165	N	*Staphylococcus aureus*	*Staphylococcus aureus*
16	M	65	C, S	160	N	*Staphylococcus aureus*	
17	M	52	L, S	185	N	*Staphylococcus aureus*	*Staphylococcus aureus*

M, Male; F, Female; C, Cervical vertebra; T, Thoracic vertebra; L, Lumbar vertebra; PM, Psoas Major; N, No prior operations; S, Surgical site of infection.

## Discussion

In statistical analysis, the positive rate of bacterial culture from tissue samples taken during surgery was found to be 47.06% (8/17), which is consistent with previous studies (10–13). This indicates that our method of obtaining samples and conducting cultures is reliable, but also suggests that the positive rate of bacterial culture from intraoperative tissue samples is not very optimistic. In this study, our team innovatively applied NPWD technology to the treatment of spinal infections, resulting in a total drainage volume of 186.47 ± 29.44 mL, which is significantly higher than the drainage volume obtained from non-NPWD (14). Furthermore, the positive rate of bacterial culture was increased to 82.4%, which is a significant improvement. This is crucial because spinal infections have a poor prognosis, with a reported fatality rate of approximately 6% and a disability rate of around 27% (15, 16)^.^ If left untreated, this condition can have serious consequences for the patient’s life and lead to various complications (15, 16). Antibiotics are the main form of treatment for spinal infections, whether conservative or surgical methods are used (1, 17). However, in cases where sepsis is present or the cause of the infection is unknown, it is recommended to start empirical antibiotic treatment. The suggested duration of antibiotic treatment for bone tissue infections is 3–8 weeks, while for non-specific spinal infections, it is recommended to use antibiotics for at least 6 weeks (18). Even with such a long period of antibiotic treatment, the effectiveness may be compromised without bacterial culture and sensitivity results (19). Therefore, improving the positive rate of bacterial culture is crucial, and our research results are of great help in this regard.

Several factors can affect the likelihood of detecting microbial cultures, including prior antibiotic use, the amount of infected tissue obtained, specimen collection method, and sample storage. The probability of identifying pathogenic bacteria decreases significantly if antibiotics were used before sample collection, dropping from 70 to 30%. Therefore, it is recommended to identify the specific pathogenic microorganisms before starting antibiotic treatment if possible. While infections like spinal *tuberculosis* or *brucellosis* can be detected through serological tests, non-specific infections such as *Staphylococcus aureus* or *Escherichia coli* have a lower positivity rate in blood cultures, typically ranging from 40 to 60% (10). Pus cultures have a wider range of positivity rates, varying from 30 to 91% (11–13), but not all patients produce pus.

The traditional tissue culture method has a higher positive rate compared to blood culture, but it is still not satisfactory. Research has shown that the positive rate of specimens obtained through minimally invasive extraction, incision extraction, or ordinary drainage is similar (20). In our case data, the positive rate of tissue samples obtained during surgery and sent for bacterial culture was 47.06% (8/17), which is consistent with the literature. This could be due to the small amount of pathogenic bacteria present and the use of antibiotics by many patients before treatment. Furthermore, catheter drainage does not significantly increase the positive rate of drainage culture compared to other specimen cultures. In our study, a significant increase in the positive culture rate was observed. This could be attributed to the negative pressure generated by Negative Pressure Wound Therapy (NPWT), which continuously extracts tissue exudates, pus, and other substances. The pressure is evenly distributed on the surface of polyvinyl alcohol (PVA), creating comprehensive drainage. NPWT is able to remove water from bacterial biofilms and disrupt the bacterial growth environment (21). Additionally, NPWT can attract immune cells such as macrophages and white blood cells to the infected site, enhancing their activity and phagocytosis (22). With the continuous negative pressure effect, bacteria are drained into a collection bottle outside the body, meeting the cultivation requirements.

In conclusion, antibiotics are essential for treating spinal infections. The traditional method of blood culture and puncture specimen bacterial culture has a low success rate. However, using negative pressure drainage to collect drainage fluid for cultivation can significantly increase the positive rate and aid in the diagnosis and treatment of spinal infection. Despite this, this study was a single center retrospective study with a limited sample size, so further research with a larger sample size is necessary.

## Data Availability

The original contributions presented in the study are included in the article/supplementary material, further inquiries can be directed to the corresponding author.

## References

[1] Berbari EF Kanj SS Kowalski TJ Darouiche RO Widmer AF Schmitt SK. 2015 Infectious Diseases Society of America (IDSA) clinical practice guidelines for the diagnosis and treatment of native vertebral osteomyelitis in Adultsa. Clin Infect Dis. (2015) 61:e26–46. doi: 10.1093/cid/civ482, PMID: 26229122

[2] Gasbarrini AL Bertoldi E Mazzetti M Fini L Terzi S Gonella F. Clinical features, diagnostic and therapeutic approaches to haematogenous vertebral osteomyelitis. Eur Rev Med Pharmacol Sci. (2005) 9:53–66. PMID: 15852519

[3] Qi CZ Yang Y. Variation of spinal infection in a hospital of Jinan city over the past 18 years. J Orthop. (2020) 28:2154–7. doi: 10.3977/j.issn.1005-8478.2020.23.10

[4] Khan IA Vaccaro AR Zlotolow DA. Management of vertebral diskitis and osteomyelitis. Orthopedics. (1999) 22:758–65. doi: 10.3928/0147-7447-19990801-07 10465488

[5] Butler JS Shelly MJ Timlin M Powderly WG O’Byrne JM. Nontuberculous pyogenic spinal infection in adults. Spine. (2006) 31:2695–700. doi: 10.1097/01.brs.0000244662.78725.37, PMID: 17077738

[6] Bydon M de la Garza-Ramos R Macki M Naumann M Sciubba DM Wolinsky JP. Spinal instrumentation in patients with primary spinal infections does not lead to greater recurrent infection rates: an analysis of 118 cases. World Neurosurg. (2014) 82:e807–14. doi: 10.1016/j.wneu.2014.06.014, PMID: 24937598

[7] Pupaibool J Vasoo S Erwin PJ Murad MH Berbari EF. The utility of image-guided percutaneous needle aspiration biopsy for the diagnosis of spontaneous vertebral osteomyelitis: a systematic review and meta-analysis. Spine J. (2015) 15:122–31. doi: 10.1016/j.spinee.2014.07.003, PMID: 25058561

[8] Omran K Ibrahim AH. Outcome of transforminal lumbar thorough debridement, decompression, and Spondylodesis technique in treatment of 25 patients with pyogenic spondylodiscitis. World Neurosurg. (2019) 124:e197–207. doi: 10.1016/j.wneu.2018.12.068, PMID: 30610978

[9] Wang J Yang Y Xing W Xing H Bai Y Chang Z. Safety and efficacy of negative pressure wound therapy in treating deep surgical site infection after lumbar surgery. Int Orthop. (2022) 46:2629–35. doi: 10.1007/s00264-022-05531-w, PMID: 35931831

[10] Renfrew DL Whitten CG Wiese JA el-Khoury GY Harris KG. CT-guided percutaneous transpedicular biopsy of the spine. Radiology. (1991) 180:574–6. doi: 10.1148/radiology.180.2.2068332, PMID: 2068332

[11] Gras G Buzele R Parienti JJ Debiais F Dinh A Dupon M. Microbiological diagnosis of vertebral osteomyelitis: relevance of second percutaneous biopsy following initial negative biopsy and limited yield of post-biopsy blood cultures. Eur J Clin Microbiol Infect Dis. (2014) 33:371–5. doi: 10.1007/s10096-013-1965-y, PMID: 24057139

[12] Grammatico L Baron S Rusch E. Epidemiology of vertebral osteomyelitis (VO) in France: analysis of hospital-discharge data 2002–2003. Epidemiol Infect. (2008) 136:653–60. doi: 10.1017/S0950268807008850, PMID: 17568478 PMC2870846

[13] Kim BJ Lee JW Kim SJ Lee GY Kang HS. Diagnostic yield of fluoroscopy-guided biopsy for Infectious spondylitis. AJNR Am J Neuroradiol. (2013) 34:233–8. doi: 10.3174/ajnr.A3120, PMID: 22627798 PMC7966322

[14] Hao X Qi CZ Lin L. Comparing and contrasting the effects of vacuum sealing drainage and general negative pressure drainage for the treatment of thoracolumbar infection. Acta Acad Med Weifang. (2024) 46:64. doi: 10.16846/j.issn.1004-3101.2024.01.012

[15] Nolla JM Ariza J Gómez-Vaquero C Fiter J Bermejo J Valverde J. Spontaneous pyogenic vertebral osteomyelitis in nondrug users. Semin Arthritis Rheum. (2002) 31:271–8. doi: 10.1053/sarh.2002.29492, PMID: 11836660

[16] Torda AJ Gottlieb T Bradbury R. Pyogenic vertebral osteomyelitis: analysis of 20 cases and review. Clin Infect Dis. (1995) 20:320–8. doi: 10.1093/clinids/20.2.320, PMID: 7742437

[17] Nagashima H Tanishima S Tanida A. Diagnosis and management of spinal infections. J Orthop Sci. (2018) 23:8–13. doi: 10.1016/j.jos.2017.09.016 29066036

[18] Bernard L Dinh A Ghout I Simo D Zeller V Issartel B. Antibiotic treatment for 6 weeks versus 12 weeks in patients with pyogenic vertebral osteomyelitis: an open-label, non-inferiority, randomised, controlled trial. Lancet. (2015) 385:875–82. doi: 10.1016/S0140-6736(14)61233-2, PMID: 25468170

[19] Jeong SJ Choi SW Youm JY Kim HW Ha HG Yi JS. Microbiology and epidemiology of Infectious spinal disease. J Korean Neurosurg Soc. (2014) 56:21–7. doi: 10.3340/jkns.2014.56.1.21, PMID: 25289121 PMC4185315

[20] Kim CJ Kang SJ Choe PG Park WB Jang HC Jung SI. Which tissues are best for microbiological diagnosis in patients with pyogenic vertebral osteomyelitis undergoing needle biopsy? Clin Microbiol Infect. (2015) 21:931–5. doi: 10.1016/j.cmi.2015.06.021, PMID: 26119720

[21] Lianhua Y Yunchao H Guangqiang Z Kun Y Xing L Fengli G. The effect of Iatrogenic*staphylococcus epidermidis*intercellar adhesion operon on the formation of bacterial biofilm on polyvinyl chloride surfaces. Surg Infect. (2014) 15:768–73. doi: 10.1089/sur.2013.129, PMID: 25402758 PMC4268582

[22] Xu J Wang QY Li W. Autologous platelet-rich gel and continuous vacuum sealing drainage for the treatment of patients with diabetic foot ulcer. Medicine. (2019) 98:e17928. doi: 10.1097/MD.0000000000017928, PMID: 31725645 PMC6867736

